# Prevalence and antimicrobial-resistant patterns of *Pseudomonas aeruginosa* among burn patients attending Yekatit 12 Hospital Medical College in Addis Ababa, Ethiopia

**DOI:** 10.1371/journal.pone.0289586

**Published:** 2024-03-07

**Authors:** Fedasan Alemu Abdi, Abdi Negash Motumma, Alem Abrha Kalayu, Woldearegay Erku Abegaz

**Affiliations:** 1 Department of Medical Laboratory Science, College of Health Sciences, Salale University, Oromia, Ethiopia; 2 Department of Microbiology, Immunology, and parasitology, School of Medicine, Addis Ababa University, Addis Ababa, Ethiopia; North Carolina State University, UNITED STATES

## Abstract

**Background:**

Burns are one of the most common forms of trauma globally. *P*. *aeruginosa* plays a prominent role as an etiological agent among burn patients. There is a paucity of information about the prevalence and antimicrobial resistance patterns of *P*. *aeruginosa* among burn patients in Ethiopia. Hence, this study was designed to assess the prevalence and antimicrobial-resistant patterns of *P*. *aeruginosa* among burn patients attending Yekatit 12 Hospital Medical College in Addis Ababa, Ethiopia.

**Methods:**

Hospital-based cross-sectional study was conducted at Yekatit 12 Hospital Medical College among burn patients from November 2020 to April 2021. Identification of *P*. *aeruginosa* was performed using Culture, Biochemical tests, and, Gram staining. Antimicrobial resistance testing was done using the Kirby-Bauer disc diffusion method. Logistic regression was computed to determine associated factors.

**Results:**

From 210 burn wound cultures, 27 (12.86%) were found positive for *P*. *aeruginosa*. All the isolates showed greater than 70% susceptibility to the tested antibiotics except Gentamycin, Ceftazidime, and, Ciprofloxacin. In addition, 33.33% of *P*. *aeruginosa* isolates were multidrug-resistant. Admission type, Hospital stay time and Total body surface area (TBSA) had a statistically significant association (all with P-value <0.05) with the acquisition of *P*. *aeruginosa* infection.

**Conclusion:**

Overall, the prevalence of *P*. *aeruginosa* isolates among burn patients is almost 13%. Most *P*. *aeruginosa* isolates were sensitive to Imipenem, while they were most resistant to Gentamycin. One-third of *P*. *aeruginosa* were multidrug-resistant. This suggests the need to monitor the treatment of infection with the pathogen to limit the possibility of the emergence of multidrug-resistant isolates in burn centers.

## Background

Burns are one of the most common and devastating forms of trauma mostly caused by heat, radiation, electricity, or contact with chemicals. Burns removes the protective skin layer, which results in disruption of the normal skin barrier accompanied by depression of immune responses, as a result of which the body is generally exposed to numerous potential pathogens. Microbial infection after burns, where a large part of the skin is damaged, is a very serious complication that is often the principal cause of patients’ death [1, 2]. Infection in burn wounds is the major cause of disability and mortality affecting all ages in both developed and developing countries [3].

The burn wound surfaces contain a large amount of necrotic tissue and a protein-rich environment that provides a favorable niche for microbial colonization and proliferation [4]. Although the burn wound area is sterile immediately following thermal/burn injury, a complex and changing microbial ecology rapidly develops thereafter within an average of 5 to 7 days [5].

*P*. *aeruginosa* is a commonly known opportunistic pathogen frequently causing serious infection and complications in burned patients throughout the world, which accounts for about 45% of mortality among these patients [6, 7]. The presence of dead, denatured tissues and a moist environment makes the burn wound vulnerable to infection by *P*. *aeruginosa* [8]. Additionally, a breach in the protective skin barrier, reduced immunity, and prolonged hospital stay are an important factor responsible for infection of burn wounds with such opportunistic pathogens, especially with multi-drug resistant (MDR) *P*. *aeruginosa* [9].

Infections caused due to *P*. *aeruginosa* are difficult to cure and challenging because this organism has a natural susceptibility to a very limited number of antimicrobial agents and often require combination therapy because high rates of resistance to antibiotics are associated with *P*. *aeruginosa* strains. Additionally, the genetic changes and adaptive behavior of these bacteria within the biofilm make them resistant to all known antimicrobial agents, making the *P*. *aeruginosa* infections more complicated and life-threatening [10, 11].

Several resistance mechanisms are implicated in the *P*. *aeruginosa* strains; it displays high intrinsic resistance to a wide variety of antibiotics (including Aminoglycosides, Fluoroquinolones and B-lactams), acquired resistance and adaptable resistance as indicated below (Table 1) [12].

**Table 1:** Overview of the different types of resistance exhibited by *P*. *aeruginosa* [12].

Class of Resistance	Stable*	Inheritable	Dependence on environment	Mechanisms	Examples of genes involved
Intrinsic	+	+	_	Low outer membrane permeability, β-lactamase production and efflux pump overexpression	*Crc*,*lon*,*psra*
Acquired	+	+	_	Horizontal transfer, mutations leading to reduced uptake and efflux pump overexpression	*ampD*, *gyrA*, *nalA*, *nfxB*, *cbrA*,*MBLs*,
Adaptive	-	-	+	Gene expression changes including β-lactam and efflux pump overexpression owing to factors triggering expression of regulatory genes	*ampC*, *mexZ*, *phoQ*

NB: *, +: property applies; -, not a property of this form of resistance.

The one main mechanism is the resistance to carbapenems, which are widely used as the most important drugs for the treatment of *P*. *aeruginosa*-associated infections. However, resistance to these compounds has also become a growing therapeutic problem. It is also resistant to all or almost all β-lactam antibiotics, aminoglycosides, and quinolones: namely, cefepime, ceftazidime, imipenem, meropenem, piperacillin-tazobactam, ciprofloxacin, and levofloxacin. A combination of resistance mechanisms is usually present. The World Health Organization has ranked carbapenem-resistant *P*. *aeruginosa* as a critical target (priority 1) for which new antimicrobial agents are needed. Unfortunately, with the increasing use of broad-spectrum antibiotics, the incidence of multi-drug resistant *P*. *aeruginosa* is increasing, and the clinical treatment of these infections is becoming even more challenging [13–16].

This bacterium can simply develop resistance to all conventional antipseudomonal antimicrobials through one-of-a-kind intrinsic and acquired resistance mechanisms. This bacterium commonly demonstrates multiple resistant isolates, which represent a serious threat to public health due to their limited therapy and leading to morbidity and mortality [17]. Furthermore, highly problematic in burn situations is both the spread of *P*. *aeruginosa* from one patient to another and the persistence of this strain in patients throughout several courses of antibiotic treatment, which were administered to treat *P*. *aeruginosa* and non-*Pseudomonas* infections [10, 18]. Therefore, currently *P*. *aeruginosa*, antibiotic resistance is an increasing problem globally and raises serious concerns [18, 19].

## Materials and methods

### Ethics approval and consent to participate

This study has been conducted in accordance with the Helsinki Declaration and Ethiopian research regulations. Ethical clearance was obtained from the Departmental Research Ethics and Review Committee of Addis Ababa University by Protocol Number: DRERC/005/2020. An Official letter was written from the Department of Microbiology, Immunology, and Parasitology to Yekatit 12 Hospital Medical College and the hospital granted permission for sample collection. Participants were informed of the purpose of the study, risks associated with the study, confidentiality of personal data, and their right to take part in the study. After that, we obtained a written informed consent form from adult study participants, whereas an assent form was obtained from study participants less than 18 years of age and, in addition to that, a consent form was also obtained from their parents or legal guardians to participate in this study. Finally, a specimen was collected from all study participants. Laboratory results of study participants were communicated with their respective physicians for better management.

### Study design, study settings and period

A Hospital-based cross-sectional study was conducted at Yekatit 12 Hospital Medical College, Addis Ababa City, Ethiopia, from November 2020 to April 2021. The Hospital is located at Arada Sub-City in Addis Ababa and is a teaching hospital under Addis Ababa City Administration Health Bureau. The hospital has around 272 beds offering different medical services and has a high number of burn beds with a dedicated burn center that provides services for a high number of adult and pediatric burn patients. All the patients admitted to the Burns Center at Yekatit 12 Hospital Medical College were treated with a national standardized protocol that includes critical care, surgical debridement of wound, topical antimicrobial application, regular wound cleaning and wound closure (autograft) as soon as possible, rehabilitation, and antibiotics given based on culture results.

### Study population and sampling techniques

Burn patients attending Pediatric and Adult Inpatient department (IPD) and Outpatient department (OPD) burn wards who had wound infections and who fulfilled the inclusion criteria were involved in the study. By using the non-probability, Convenience sampling technique, 210 burn patients were involved in the study.

### Sample size

The sample size was calculated based on a single sample size estimation by considering the report of 14.5% *P*. *aeruginosa* prevalence among burn wound patients from a previous study done in South Africa [20], 95% level of confidence (α = 0.5), with the tolerable error of 5% (d = 0.05) and Assumption of 10% non-response rate.


$$\text{n}=\frac{{\text{Z}}^{2}\text{P}\left(1-\text{P}\right)}{{\text{d}}^{2}}=191+10\%=210$$

### Specimen collection and processing

Burn wound swabs were collected once by using a sterile cotton swab after cleansing the wounds with normal saline before obtaining swab specimens. Following collection, the swabs were placed into a sterile test tube with a screw cup and were transported to the microbiology laboratory within 30 minutes.

In the Microbiology laboratory at Yekatit 12 Hospital Medical College, all the burn swab samples were inoculated onto Mac Conkey agar and blood agar plates (BAP). The plates were incubated at 35–37°C for 24–48 hours and examined for bacterial growth. Different biochemical tests were performed from pure culture colonies for the final identification of *P*. *aeruginosa* isolates, such as catalase, oxidase, motility, citrate, triple sugar iron, urea, lysine iron agar, and indole tests.

Finally, antimicrobial susceptibility testing was done using the Kirby Bauer disk diffusion method on Mueller-Hinton agar. Antibiotics regularly available and frequently prescribed in the study area were included in the study. These include—Ceftazidime (30μg), Ciprofloxacin (5 μg), Gentamicin (10 μg), Tobramycin (10μg), Cefepime (30μg), Meropenem (10μg), Imipenem (10μg), Amikacin (30 μg) and Piperacillin-tazobactam (30 μg).

The results were then interpreted as Sensitive (S), Susceptible, increased exposure (SIE) or Resistant (R) according to CLSI guidelines (2019) [21].

### Data quality assurance

Data quality was ensured through the use of standardized data collection materials, proper training of data collectors before the start of data collection, and intensive supervision during data collection by the principal investigator. The performance of the media and antibiotic discs were evaluated using positive controls; i.e. American Type Culture Collection (ATCC) 27853 references a strain of *P*. *aeruginosa*.

### Data analysis and interpretation

Data was entered and analyzed using SPSS version 25.0. Descriptive statistics, frequency, and percentage were used to describe the study participants. Bivariate and multivariate logistic analyses were performed to assess the association of the factors associated with *P*. *aeruginosa* infection. A p-value of <0.25 in the univariate analysis was the criterion for including variables in the multivariable analysis. In all cases P-value, less than 0.05 was considered statistically significant. Results were presented using figures and tables.

## Results

### Sociodemographic and clinical data characteristics of burn patients

During the five months of the study period, 210 study participants with burn wound infections were included. Burn wound swabs were collected from study participants admitted to burn IPDs 110 (52%) and burn OPDs 100 (48%). Among these, (53.3%) were females, and the age range was 1 to 85 years (Mean 20 years and Median 18years). The majority of the study participants (43.8%) were in the age group of 0–15 years. Pertaining to the causes of burn injury, the majority (46.7%) sustained burn through scald followed by open flame (37.6%). The anatomical site of burn injury was mostly extremities (67.1%) followed by the Head and Neck (9.5%). Regarding the depth of burn wounds, the majority (56.2%) suffered from a 2^nd^ degree burn followed by a 1^st^ degree burn level of (29.5%).

Regarding participants’ comorbidity, only 35 cases (16.7%) had different diseases when admitted to the hospital, with whom mental problem/Epilepsy accounted for (40%) and Diabetic Mellitus (DM) for (34.29%). Concerning TBSA, the percentage of burns ranged from 3% to 45%; the medium value is 13%. Almost half of the patients had TBSA <10%, which accounted for (41%), followed by 10–19% TBSA (33.33%). Concerning Hospital Stay time, (91.4%) of the patients had a hospital stay time between 2-30days, and only 18 cases (8.6%) had a hospital stay time above one month (Table 2).

**Table 2:** Summary of socio-demographic and clinical data characteristics of burn patients at Yekatit 12 Hospital Medical College, Addis Ababa- Ethiopia, 2021.

Variables	Category	Frequency	Percent (%)
**Age**	0–15	92	43.8
	16–40	90	42.8
	41–60	22	10.5
	>60	6	2.9
**Sex**	Male	98	46.7
	Female	112	53.3
**Educational level**	No formal education	80	38.1
	Elementary	78	37.1
	High school	31	14.8
	College and above	21	10
**Residence**	Urban	130	61.9
	Rural	80	38.1
**Occupation**	Government employed	18	8.6
	Self employed	37	17.6
	Daily laborer	35	16.7
	Student	50	23.8
	House wife	38	18.1
	Others	32	15.2
**Admission type**	OPD burn wards	100	48
	IPD burn wards	110	52
**Etiology of burns**	Scalds	98	46.7
	Open flame	79	37.7
	Chemical	8	3.8
	Electrical	19	9
	Others	6	3.8
**Anatomical site**	Extremities	141	67.1
	Trunk	5	2.4
	Head & Neck	20	9.5
	Head, Neck &Extremities	15	7.1
	Head, Neck, Perineum &Extremities	11	5.3
	Extremities and Perineum	15	7.1
	Whole body Parts	3	1.5
**Level of burn**	1^st^ degree	62	29.5
	2^nd^ degree	118	56.2
	3^rd^ degree	22	10.5
	4^th^ degree	8	3.8
**Co-morbidity**	Epilepsy and/or other mental health disorders	14	40
	DM	12	34.29
	HIV	5	14.29
	Others	4	11.42
**Total body surface area(TBSA) involved in the burn**	<10%	86	41
	10−19%	70	33.3
	20−29%	38	18.1
	≥30%	16	7.6
**Hospital stay Time**	2-10days	86	41
	11-20days	58	27.6
	21-30days	48	22.8
	>30days	18	8.6

### Distribution and prevalence of *P*. *aeruginosa* among burn patients

Among the 210 burn wound swabs, 27 (12.86%) yielded a positive result for *P*. *aeruginosa*. The *P*. *aeruginosa* isolation rate in the 0–15 age category was higher (66.7%) among all age groups. Moreover, 14 (51.85%) of the isolates were females. Most of the *P*. *aeruginosa* isolates, 24(89%), were obtained from burn IPD and only 3 isolates were from burn OPD (11%).

**Fig 1:**
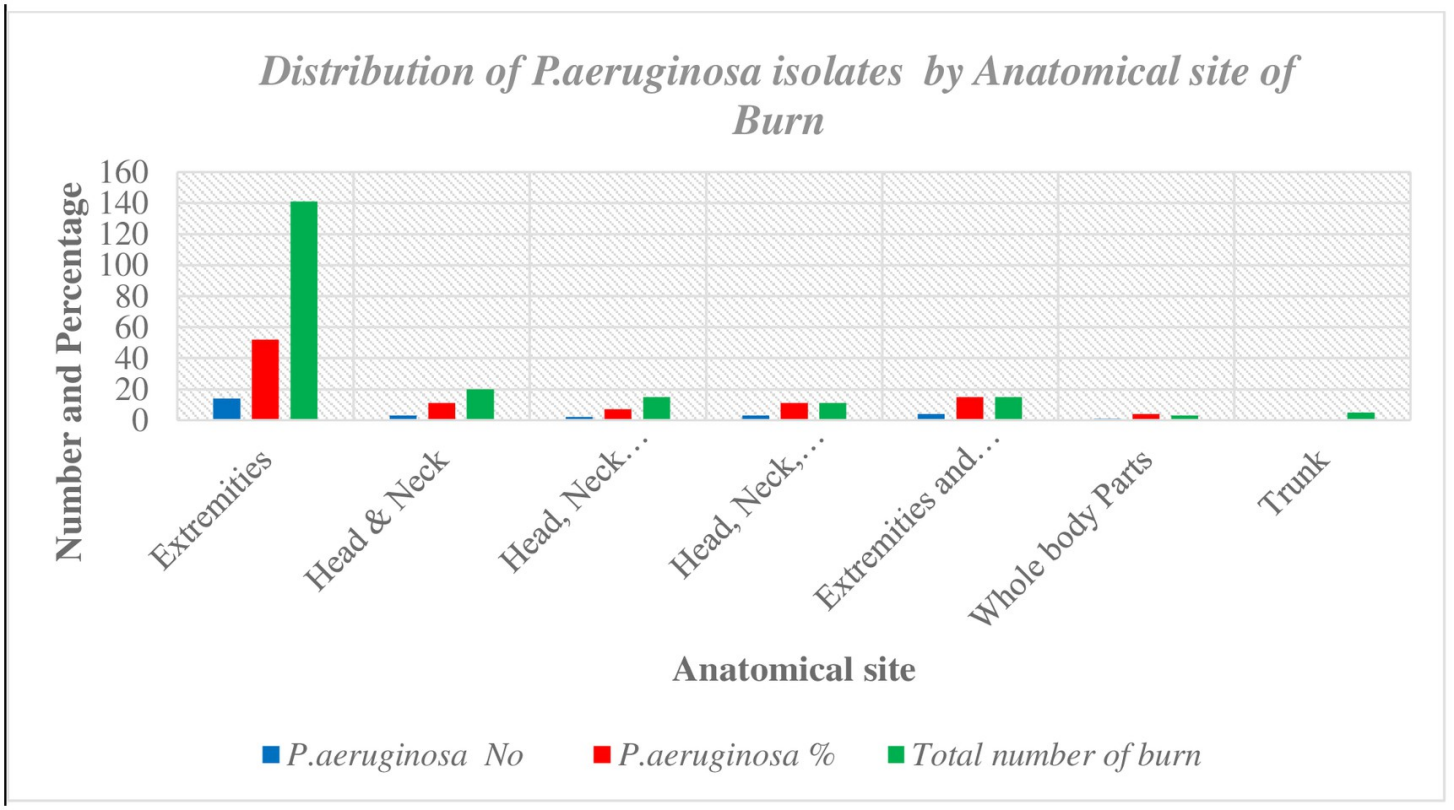
Distribution of *P*. *aeruginosa* isolates by anatomical site of burns among burn patients at Yekatit 12 Hospital Medical College burn center, Addis Ababa- Ethiopia, 2021.

*P*. *aeruginosa* isolation rate was highest (15/27) from burn injury due to scalds followed by open flame (7/27; 26%), electrical (2/27; 7%), and chemical (1/27; 4%). Likewise, the proportion of *P*. *aeruginosa* was highest in burn injury from Extremities anatomical site (14/27; 52%) followed by Extremities and Perineum (4/27; 15%), (Fig 1).

Regarding the depth of burn wounds, the proportion of *P*. *aeruginosa* was highest in 2^nd^ degree burns (16/27; 59.3%) followed by 4^th^ degree burns (5/27; 18.5%). There was also the highest *P*. *aeruginosa* isolation rate (11/27; 40.7%) from the TBSA category group of ≥30%, followed by 20–29% (9/27; 33.3%). The *P*. *aeruginosa* isolation rate for the duration of hospital stay showed the highest proportion among those participants with 21–30 days (22/27; 81.5%), which was then followed by > 1 month (3/27; 11.1%), 11–20, and 2–10 days each counted for 1/27 isolate (4%).

### Antimicrobial resistance patterns of *P*. *aeruginosa* isolate from burn patients

**Fig 2:**
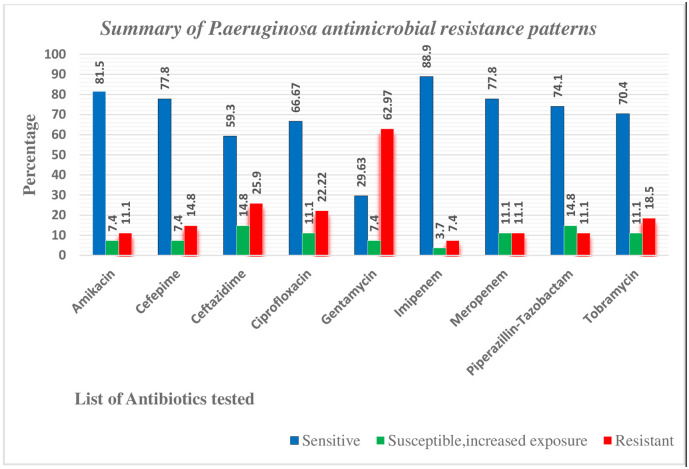
Summary of antibiotic resistance patterns of *P*. *aeruginosa isolates* among burn patients at Yekatit 12 Hospital Medical College burn center, Addis Ababa- Ethiopia, 2021.

Antimicrobial susceptibility testing was carried out for all of the 27 *P*. *aeruginosa* isolates using the Kirby-Bauer disk diffusion method. The most effective antibiotic was found to be Imipenem with 88.90% sensitivity, followed by Amikacin (81.50%) whereas, Gentamycin was the least effective antibiotic with a 62.97% resistance rate, (Fig 2). A total of (33.33%) *P*. *aeruginosa* isolates were multidrug-resistant, i.e., resistant to at least one drug from three or more antibiotic classes. From the total of 27 isolates of *P*. *aeruginosa*, only 4 isolates (15%) were 100% sensitive to all drugs tested and conversely, no isolate was found resistant to all the antibiotics tested.

### Possible factors associated with *P*. *aeruginosa* among infection burn patients

The results of multiple logistic regression showed that only Admission type (positive association with Inpatient department (IPD) wards; P = 0.031), Total body surface area of ≥30% (P = 0.005) and Hospital stay time >1month (0.011) had a statistically significant association of *P*. *aeruginosa* isolation rate from burn wound. Patients with Burn area (TBSA) of ≥30% were 6.8 times more likely to have *P*. *aeruginosa* isolates whereas patients with Burn area (TBSA) of 20–29% were 3.1 times more likely to have *P*. *aeruginosa* isolates compared to those with TBS of <10%. Similarly, multiple logistic regression analysis showed that as the duration of hospital admission increased, the rate of *P*. *aeruginosa* isolation also increased, with patients with hospital admission >1months being 4.6 times more likely to have *P*. *aeruginosa* isolates compared to those with 2–10 days hospital admission (Table 3).

**Table 3:** Bivariate and multivariate analysis that shows the relationship between associated factors and prevalence of *P*. *aeruginosa* among burn patients at Yekatit 12 Hospital Medical College burn center, Addis Ababa- Ethiopia, 2021.

Variables	Category	COR	P-Value	AOR	P-Value
		(95% CI)		(95% CI)	
**Age**	0–15	(Ref.)			
	16–40	2.37(0.24–23.22)	0.46	1.47(0.1–33)	0.79
	41–60	4.20(0.22–79.32)	0.24	1.48(0.15–85)	0.32
	>60	4.8(0.1–7.5)	0.34	4.05(0.56–29)	0.17
**Admission type**	OPD burn wards	(Ref.)			
	IPD burn wards	9.2(2.6–31)	0.001*	7.47(1.15–5.67)	0.031*
**Cause of burns**	Scalds	(Ref.)			
	Open flame	1.5(0.15–11.09)	0.20	2.9(4.3–201.7)	0.33
	Chemical	1.3(0.32–73.5)	0.83	3.1(0.2–39)	0.74
	Electrical	1.8(0.7–48)	0.59	2.3(1.17–44.6)	0.42
	Others	0.3(0.03–42.4)	0.42	0.18(0.5–62)	0.34
**Anatomical site**	Trunk	(Ref.)			
	Extremities	1.4(0.81–14.32)	0.095	1.23(0.78–39)	0.74
	Whole body Parts	6.5(0.05–11.65)	0.84	9(1.10–45)	0.58
	Extremities and Perineum	1.02(0.18–5.95)	0.97	1.8(1.2–87)	0.44
	Head and Neck	2.12(0.35–12.95)	0.41	2.5(0.01–54)	0.78
	Head, Neck & Extremities	2.44(0.3–17.90)	0.38	4.2(0.14–73)	0.24
	Head, Neck, Extremities &Perineum	1.4(1.2–25)	0.56	3.3(1.7–45)	0.78
**Level of burn**	1^st^ degree	(Ref.)			
	2^nd^ degree	1.63(2.31–48)	0.002*	1.02(0.81–12.90)	0.98
	3^rd^ degree	3.56(1.6–69)	0.014*	1.2(0.9–42.3)	0.29
	4^th^ degree	10.2(5.2–20.68)	0.018*	3.7(0.1–5.95)	0.82
**Co-morbidity**	HIV	(Ref.)			
	DM	0.3(0.03–3.40)	0.33	0.2(0.01–1.24)	0.63
	Epilepsy and/or other mental health disorders	0.36(0.02–7.3)	0.51	0.7(0.01–5.53)	0.21
	Others	0.14(0.4–20.7)	0.15	NA	
**Total body surface area(TBSA)**	<10%	(Ref.)			
	10–19%	3.9(1.6–37.6)	0.001*	1.50(0.26–17.70)	0.003*
	20–29%	7.73(0.2–26)	0.002*	3.1(0.19–17.5)	0.006*
	≥30%	8.3(0.58–16.47)	0.000*	6.8(1.2–10.6)	0.005*
**Hospital stay Time**	2-10days	(Ref.)			
	11-20days	1.40(0.21–10.80)	0.012*	2.4(0.19–16)	0.021*
	21-30days	5.9(0.6–6.04)	0.017*	3.95(0.1–25.6)	0.050*
	>1months	6.7(0.41–10.9)	0.001*	4.6(2.9–21.5)	0.011*

**AOR:** Adjusted Odds ratio, **COR**: Crude Odds ratio, **CI**: Confidence interval, **Ref**: Reference category, **Bold p-value**: Significant association

## Discussion

### Prevalence of *P*. *aeruginosa* among burn patients

This study enrolled 210 burn wound patients, of whom at least (12.86%) were infected with *P*. *aeruginosa*, which is in agreement with findings from previous similar studies done in India, and Turkey, each with the rate of 12% [22, 23], Tanzania,12.6% [24], Kenya,13.7% [25] and South Africa, 14.5% [20]. On the other hand, the 12.86% isolation rate from this study is higher than those reported in other previously done studies both in Ethiopia and elsewhere in the world:—for example,4.8% in Ethiopia [26] and 6.25% in Nepal [27].Contrary to this, considerably higher *P*. *aeruginosa* isolation rates were reported from various countries including 39.6% from Ethiopia [28], 21% from China [29], 22.4% from Malaysia [19], 24.9% from Pakistan [30], 27% from Iraq [31], 30.2% from Ghana [32], 46.5% from Yemen [33], 55% from India [34], 57% from Iran [35] and 62.7% from Nigeria [36]. Such variations in *P*. *aeruginosa* isolation rates from burn wounds between studies could be due to one or a combination of the following reasons: (1) the type of inclusion criteria, (2) the use of selective/differential media for culturing the target organism, (3) sampling protocols, and (4) differences in facilities of health care providing institutions including presence or absence of dedicated centers for handling burn injuries.

### Antibiotics resistance patterns of *P*. *aeruginosa* among burn patients

The majority of antibiotics tested in this study, namely, -Imipenem, Meropenem, Amikacin, Piperacillin-tazobactam, Tobramycin and Cefepime were found to be the most active antimicrobial agents against *P*. *aeruginosa* isolates. On the contrary, Gentamycin was the most resisted antibiotic (with 63% resistance and only 30% sensitivity rate) followed by ceftazidime (with 26% resistance and 59% sensitivity rate) and ciprofloxacin (with 22% resistance and 67% sensitive rates), which might indicate that these drugs have been used frequently in the burn treatment center of the study site.

According to a systematic review and meta-analysis done in China, there was an increasing trend of *P*. *aeruginosa* resistance to a common antimicrobial agent of wound-isolated among burn patients: This study indicates that *Gentamicin had the highest pooled resistance rate (56%)while meropenem had the lowest pooled resistance rate (29%)* [29]. Another meta-analysis done in Iran among burned patients also showed that the most common resistance was seen against ceftazidime (66.9%), followed by ciprofloxacin *(52*.*9%) and cefepime (52*.*3%) whereas*,*54*.*9% of P*. *aeruginosa* isolate were resistant to *imipenem* [37].

Comparable resistance rates for *P*. *aeruginosa* isolates were observed against Gentamycin in Kenya [25], India [34] and Pakistan [38]. Higher Gentamycin resistance by *P*. *aeruginosa* appears to also be a common phenomenon in some countries, like India (84%) [9], Yemen (87%) [33], Iraq (88.5%) [31], and Malaysia (94.3%) [19]. However, some other studies reported lower resistance levels; for example, 9.2% in South Africa [20], 13% in Ghana [32], 24.7% in Nigeria [8], 36% in Turkey [23] and 44.7% in Kenya [25].

It becomes clear, from the findings of this study, that Imipenem was the most effective antibiotic with only a 7.4% resistance rate, which is consistent with reports from studies done in South Africa [20], Nigeria [8] and Pakistan [38]. Unlike these observations, however, other studies have reported variable but considerably higher rates of Imipenem resistance: 17.5% in Nigeria [8], 31.7% in Kenya [25], 32% in Iraq [31], 46% in Turkey [23], 61% in India [9], 66.7% in Pakistan [30], 73.9% in Malaysia [19] and 94.7% in Iran [39]. Probably this drug is being used heavily in these latter countries.

The next most effective antibiotics were Amikacin, followed by Meropenem & Piperacillin-Tazobactam each with 11.11% resistance. The same low rate of resistance (about 11%) against Amikacin was also documented in other studies including Yemen [33], Ghana [32], Kenya [25], and South Africa [20]. However, much higher resistance rates were reported from elsewhere for Amikacin: 21% in Turkey [23], 32.1% in Nigeria [8], 48.1% in Iraq [31], 50% in Malaysia [19], 73.2% in India [9], 75% in Pakistan [30], and 89.4% in Iran [39].

Overall, except for Gentamycin, sensitivity to all other tested antibiotics was over 50%. Compared with the susceptibility of *P*. *aeruginosa* to antimicrobials reported from the literature in several studies, *the P*. *aeruginosa* drug resistance level observed in this study against systemic antibiotics was found to be low. This might be due to limited exposures of the pathogen to the new generation broad-spectrum antibiotics tested, or due to low development of cross-resistance among *P*. *aeruginosa* isolates from the settings under the study, or due to the infrequent use of systemic antibiotics in the burn center under investigation.

On the other hand, the overall rate of MDR *P*. *aeruginosa* isolates was 33.33%, which goes in line with the result from studies done in Ethiopia 36.5% [40], China (28%) [29] and Pakistan (29.24%) [38]. But much higher than the resistance rate reported in Malaysia (5.74%) [11], India (12%) [22], Pakistan (12.2%) [30] and Kenya (12.7%) [25]. In contrast, the 33% MDR *P*. *aeruginosa* isolation rate from this study is lower than the findings from other studies elsewhere, which reported from as low as 40.7% to as high as 100% [8, 9, 27, 33, 39]. The possible explanation for such disparity might be differences in the study population, use of different antibiotic regimes, extensive use of the antimicrobial drugs studied in those settings, persistent presence of resistant strains in hospitals, cross-contamination from the laboratory environments during culturing or the quality of hygiene in the hospital environments under study.

### Possible factors associated with *P*. *aeruginosa* infection among burn patients

Bivariate and multivariate regression analyses indicated that TBSA, Level of burn, Admission type and Length of Hospital stay had statistically significant (all with P-value <0.05) association with *P*. *aeruginosa* infection. These findings are in line with reports from other similar studies done in Ethiopia [26], Nigeria [36], Netherlands [7] and Iraq [31]. Such associations may not be unexpected given that larger burn size means a greater area of the unprotected body surface and a greater chance of colonization by the pathogen because of the destruction of the surrounding structures, a condition that facilitates colonization by microorganisms [31].

Similarly, the obvious explanation for the association between length of hospital stay and *P*. *aeruginosa* infection could be that the greater time of hospitalization, patients are the most likely to contract and are colonized by the notoriously known most significant nosocomial opportunistic pathogen, *P*. *aeruginosa*. Moreover, because of time-related changes in succession of the predominant gram-positive burn wound colonizing flora during the early time to the late gram-negative bacteria (4–10 days after injury), it is expected to see significant positive associations between *P*. *aeruginosa* isolate rate and length of hospital stay, as most of the patients (21/27) from whom the pathogen was isolated comprised of those who stayed for 21-30days.

### Limitation of the study

This study was done only in a single burn center. It would have been better if it incorporated more health institutions for a better representation of study participants. Moreover, any other confirmatory test, especially the molecular test, was not done.

## Conclusion

Overall, the prevalence of *P*. *aeruginosa* among burn patients in the current study was almost 13%. The incidence of clinically significant *P*. *aeruginosa* in burn wound infection is low in this unit. However, *P*. *aeruginosa* is still the common cause of infection in our burn centers, as is the case elsewhere around the globe. It was also observed that *P*. *aeruginosa* isolates were most sensitive to Imipenem, while they were most resistant to Gentamycin, indicating that the latter is no longer potent in the treatment of *P*. *aeruginosa* among burn wound infections at Yekatit 12 Hospital Medical College. Most of the *P*. *aeruginosa* isolates had a high level of sensitivity to most examined antibiotics.

In addition, 33.3% of the isolates were multi-drug resistant, which is not only of substantial concern in the treatment center but also enlightening as to the level of MDR *P*. *aeruginosa* that one might encounter in hospital wards that are handling burn patients. Admission type (IPD burn ward), TBSA with ≥ 30%, and longer Hospital Stay time, were significantly associated with *P*. *aeruginosa* isolation.

## Abbreviations

AIDS: Acquired Immunodeficiency Syndrome
AST: Antimicrobial Sensitivity Test
CLSI: Clinical and Laboratory Standards Institute
CNS: Coagulase-Negative *Staphylococci*
DALYs: Disability-Adjusted Life-Years
DMIP: Department of Microbiology, Immunology, and Parasitology
HIV: Human Immunodeficiency Virus
LMICs: Low and Middle-income countries
LOS: Length of Hospital Stay
MDR: Multidrug-Resistant
TBSA: Total Body Surface Area
WHO: World Health Organization
Y12HMC: Yekatit 12 Hospital Medical College
